# NF-*κ*B-94ins/del ATTG Genotype Contributes to the Susceptibility and Imbalanced Th17 Cells in Patients with Immune Thrombocytopenia

**DOI:** 10.1155/2018/8170436

**Published:** 2018-07-22

**Authors:** Jie Yu, Mingqiang Hua, Xueyun Zhao, Rui Wang, Chaoqing Zhong, Chen Zhang, Ruiqing Wang, Guosheng Li, Na He, Ming Hou, Daoxin Ma

**Affiliations:** ^1^Department of Hematology, Qilu Hospital, Shandong University, Jinan, China; ^2^Department of Hematology, Weihai Municipal Hospital, Weihai 264200, China

## Abstract

**Background:**

The NLRP3 inflammasome plays important roles in the pathogenesis of autoimmune diseases. However, the role of the NLRP3 inflammasome in the pathophysiology of immune thrombocytopenia (ITP) remains unclear.

**Methods:**

RT-PCR was used to examine the polymorphism and expression of genes involved in the NLRP3 inflammasome in ITP patients. T helper cells and apoptosis of PBMC from ITP patients were analyzed by flow cytometry. The antiplatelet autoantibodies in plasma were determined by modified monoclonal antibody-specific immobilization of platelet antigens (MAIPA).

**Results:**

We found that the NF-*κ*B-94ins/del ATTG genotype contributed to the susceptibility of ITP. Furthermore, the platelet counts of ITP patients with the WW genotype or WD genotype were lower than those with the DD genotype of NF-*κ*B-94ins/del ATTG. Compared with controls, NF-*κ*B gene expression was significantly decreased and WW or WD genotype ITP patients displayed higher mRNA expression than DD individuals. Similarly, the mRNA expression of NLRP3 was also increased in the WW genotype. There was a significant gene dose effect of the percentage of Th17 cells for the WW, WD, and DD genotypes (WW < WD < DD) in the unstimulated group and no significant difference was found after being stimulated. The activation of the NLRP3 inflammasome could upregulate Th17 in ITP patients.

**Conclusion:**

The NF-*κ*B-94ins/del ATTG genotype might serve as a novel biomarker and potential target for ITP.

## 1. Introduction

Primary immune thrombocytopenia (ITP) is an acquired autoimmune disease characterized by a transient or persistent decrease of the platelet count and increased risk of bleeding [1]. Although autoreactive B lymphocytes, secreting antiplatelet antibodies, are considered as the primary immunological defect in ITP, T cellular immunity abnormalities are considered important in the pathogenesis of ITP [2, 3]. ITP has also been documented to be connected with cytokine response and dysregulation in the cytokine network [4]. Furthermore, there have been several studies reporting that the genetic variants contribute to ITP pathogenesis [5, 6].

NLRP3 (NOD-like receptor pyrin domain-containing protein 3), a well-characterized inflammasome, belongs to the innate immune system that responds to cellular stress by producing the proinflammatory cytokines IL-1*β* and IL-18. The NLRP3 inflammasome is composed of NLRP3, the adaptor protein ASC, and caspase-1. Activation of the inflammasome leads to the homotypic interaction of the PYD (pyrin domain) of NLRP3 and ASC proteins that cleaves and activates caspase-1, followed by the processing of the inactive proinflammatory cytokines IL-1*β* and IL-18 to their active forms that trigger downstream inflammatory response [7]. Caspase recruitment domain-containing protein (CARD) 8, also known as TUCAN (tumor upregulated CARD-containing antagonist of caspase nine), interacts physically with caspase-1 and negatively regulates caspase-1-dependent IL-1*β* expression and nuclear factor- (NF-) *κ*B activation. The NLRP3 inflammasome plays a role in the pathogenesis of numerous inflammatory and autoimmune diseases such as diabetes, obesity, and atherosclerosis [8, 9].

Single nucleotide polymorphisms (SNPs) are the most abundant types of DNA sequence variation in the human genome. SNPs provide a well-established method for analyzing complex gene-associated diseases and individual susceptibility for some disorders. Polymorphisms in NLRP3-associated genes are linked to common immune inflammatory diseases [10–12]. The SNP of CARD8 rs2043211 changing cysteine at codon 10 to a premature termination codon (c.30T>A; p.C10X) in this gene was found to be associated with the inflammatory activity in early rheumatoid arthritis (RA) and inflammatory bowel disease (IBD) [13, 14]. A common insertion (ins)/deletion (del) promoter polymorphism (-94ins/del ATTG polymorphism) of NF-*κ*B seems to be related to several inflammatory diseases such as ulcerative colitis (UC), Graves' disease, and psoriasis vulgaris [15–17]. Polymorphisms of the IL-18 gene have been shown to influence some chronic inflammatory diseases including diabetes, RA, systemic lupus erythematosus (SLE), and IBD [18–21]. The SNP of IL-1*β*-511T>C has been associated with promoter activity and the risk of diabetes nephropathy, RA, and UC [22–24].

There are several studies about the gene polymorphisms closely associated with the risk for ITP [5, 6]. Nevertheless, the involvement of SNPs with the NLRP3 inflammasome in ITP has not yet been demonstrated. Meanwhile, the NLRP3 inflammasome has effects on CD4+ T cell differentiation via the production of the caspase-1-dependent cytokines, IL-18 and IL-1*β*, directing Th17 responses. Th17 was an importantly proinflammatory Th cell subset, characterized by the expression of the key transcription factor RORC and secretion of IL-17 which may induce IL-17R expressed cells to produce inflammatory cytokines. Moreover, the release of IL-18 may promote the activity of IL-22, the effect factor of Th22 cells expressing the key transcription factor AHR. Our previous research reported that Th17 and Th22 cells were significantly elevated in ITP patients [25]. However, no data has been reported about the association of the NLRP3 inflammasome and T helper cells in ITP until now.

To determine the susceptibility and clinical significance of the NLRP3 inflammasome in ITP, we examined the SNPs of three NLRP3 inflammasome components and two inflammatory cytokines, including NLRP3 (rs35829419), CARD8 (rs2043211), NF-*κ*B-94ins/del ATTG, IL-18 (rs1946518), and IL-1*β* (rs16944). Then, we further carried out the functional study to explore the role of NLRP3 in Th cell development in ITP patients.

## 2. Materials and Methods

### 2.1. Subjects

A total of 403 ITP patients and 336 sex- and age-matched healthy controls were recruited prospectively in Qilu Hospital of Shandong University from July 2011 to March 2016. The diagnosis of the enrolled ITP patients was based on the American Society of Hematology guideline. Patients and healthy controls who had diabetes, pregnancy, obesity, cardiovascular disease, active or chronic infections, or connective tissue diseases were excluded in our research [26]. The response criteria and severity of the disease were defined according to the guideline [27]. The patients' characteristics were shown in Table 1. After one or two pulses of high-dose dexamethasone (HDD, 40 mg/d for 4 days), on the 14th day after treatment, we accessed the response by platelet counts and bleeding score. All the patients were followed up at least 12 months from diagnosis [27]. The ITP patients and the controls are matched for ethnicity. This study was approved by the ethics committee of Qilu Hospital of Shandong University. Informed consents were obtained from patients and controls.

### 2.2. Sample Preparations

EDTA-anticoagulated peripheral blood from newly diagnosed ITP patients before the first-line treatment was collected and centrifuged at 3000*g* for 8 minutes. Plasma supernatant was frozen at −80°C for the assay of MAIPA. Peripheral blood mononuclear cells (PBMCs) were isolated by Ficoll-Hypaque density gradient centrifugation and stored at −80°C for further analysis and functional study. Moreover, heparin-anticoagulated blood samples were collected for T helper subset analysis.

### 2.3. Genotyping of NLRP3 Inflammasome Genes

The genotyping of NLRP3 (rs35829419), IL-1*β* (rs16944), IL-18 (rs1946518), or CARD8 (rs2043211) in all subjects was performed using a standard TaqMan® allelic discrimination assay (Applied Biosystems, USA). The NF-*κ*B-94ins/del ATTG polymorphism was determined by using the forward primer: 5′-CCG TGC TGC CTG CGT T-3′, reverse primer: 5′-GCT GGA GCC GGT AGG GAA-3′ as well as probe 1: 5′-VIC-ACC ATT GAT TGG GCC-MGB-3′ and probe 2: 5′-FAM-CGA CCA TTG GGC C-MGB-3′. TaqMan SNP genotyping assay was operated on an ABI 7500 Real-Time PCR System (Applied Biosystems, Foster City, CA, USA). ABI 7500 software v1.3 and TaqMan Genotyper software were used to analyze the allele discrimination. After genotyping by PCR, five samples were randomly selected to validate the accuracy of the genotyping analysis of PCR by classical Sanger sequencing analysis.

### 2.4. The Enumeration of Megakaryocytes

Megakaryocyte quantity counting was performed according to the classical counting method. Briefly, 10 *μ*L of bone marrow aspiration was dropped onto a slide (1.5 cm × 3.0 cm) to make a smear, and then megakaryocytes were counted in the whole smear. Counting was repeated thrice and the mean was used as the megakaryocyte number of the patient.

### 2.5. Real-Time Quantitative PCR Detection

The mRNA expression of NF-*κ*B, NLRP3, IL-1*β*, IL-18, RORC, or AHR was detected by real-time quantitative PCR for 50 unselected ITP cases. Total RNA from PBMCs was isolated by the TRIzol reagent (Invitrogen, Life Technologies, Carlsbad, CA). Approximately 1 *μ*g of RNA was used to synthesize cDNA applying the PrimeScript RT Reagent Kit Perfect Real Time (Takara Bio, Japan). The quantitative PCR was performed on the LightCycler 480 II real-time PCR system (Roche, Switzerland) in accordance with the manufacturer's instruction. The primers were shown in Table 2. The real-time PCR contained, in a final volume of 10 *μ*L, 5 *μ*L of 2x SYBR Green Real-Time PCR Master Mix, 1 *μ*L of cDNA, 0.8 *μ*L of the forward and reverse primers, and 3.2 *μ*L of ddH_2_O. All experiments were conducted in triplicate. The PCR products were analyzed by melt curve analysis and agarose gel electrophoresis to determine product size and to confirm that no by-products were formed. The results were expressed relative to the number of GAPDH transcripts used as an internal control.

### 2.6. Activation of the NLRP3 Inflammasome

PBMCs were isolated from heparinized peripheral blood of ITP patients by gradient centrifugation (400 ×g for 20 min), and washed twice with sterile PBS. Then, PBMCs were resuspended in Roswell Park Memorial Institute- (RPMI-) 1640 medium supplemented with 10% heat-inactivated fetal bovine serum (Gibco, Australia) and 1% penicillin-streptomycin (Millipore, USA) at a density of 2 × 10^6^ cells/well in humidified air in 5% CO_2_ at 37°C. PBMCs were activated with 1 *μ*g/mL LPS (Sigma-Aldrich, USA) for 6 h followed 1 h later by 5 mmol/L ATP (Sigma-Aldrich, USA). Cells were then washed by PBS twice and collected to enumerate Th17 and Th22 by flow cytometric analysis. Untreated cells were used as a control. Furthermore, total RNA was extracted from the cultured PBMCs as above to determine the expression of RORC and AHR.

### 2.7. Flow Cytometry for Analysis of Th17 and Th22 Cells

Intracellular cytokines were studied by flow cytometry to identify the cytokine-producing cells. For intracellular cytokine staining, treated cells suspended with 140 *μ*L RPMI-1640 medium was incubated for 4 h at 37°C in 5% CO_2_ in the presence of 25 ng/mL of phorbol myristate acetate (PMA), 1 *μ*g/mL of ionomycin, and 1.7 *μ*g/mL of monensin (all from Multi Sciences, China). After incubation, the cells were stained with PE-Cy5-conjugated anti-CD3 and fluorescein isothiocyanate-conjugated anti-CD8 monoclonal antibodies at room temperature in the dark for 15 min to delimit CD4+ T cells because CD4 was downmodulated when cells were activated by PMA. After surface staining, the cells were stained with PE-conjugated anti-IL-17 and Fluor 660-anti-human IL-22 monoclonal antibodies after fixation and permeabilization according to the manufacturer's instructions. Stained cells were analyzed by flow cytometric analysis using a Beckman Gallios cytometer equipped with the Kaluza software (Beckman Coulter, USA).

### 2.8. Antiplatelet and Autoantibody Determination

The specific antiplatelet GP IIb/IIIa and/or GP Ib/IX autoantibodies were analyzed by modified monoclonal antibody-specific immobilization of platelet antigens (MAIPA), which was carried out as previously described in detail by Hou et al. [28].

### 2.9. Detection of Cell Apoptosis

Dexamethasone (DEX) was extensively applied as the first-line treatment of ITP patients, which may correct the imbalance of T cell subsets. For the apoptosis analysis, PBMCs cultured with or without 10 mmol/L DEX after NLRP3 activation or not were washed with PBS twice and stained with Alexa Fluor 488 Annexin V and PI using the Alexa Beyotime Cell Apoptosis Kit (Beyotime, China) according to the manufacturer's protocol. Early apoptosis (Annexin-positive and PI-negative cells) and late apoptosis (Annexin-positive and PI-positive cells) were counted, respectively, using a Beckman cytometer within 15 min after being stained. Data analysis was carried out using the Kaluza analysis software.

### 2.10. Statistical Analyses

The data was presented as median and range, and analyzed by the SPSS 17.0 software. The Chi-squared test was used to determine the Hardy-Weinberg equilibrium (HWE). We used the Chi-squared test to compare genotype and allele frequencies. Odds ratios (OR) and 95% confidence intervals (CI) were applied to evaluate the association between the gene polymorphism and ITP susceptibility. Group comparisons for the gene expression and Th frequencies were performed using the Kruskal-Wallis test. For all the compared data, a *P* value < 0.05 was considered statistically significant.

## 3. Results

### 3.1. The Polymorphism of NF-*κ*B-94ins/del ATTG Contributed to the Susceptibility of ITP

We studied 739 ethnic Northern Han Chinese subjects including a total of 403 subjects with ITP. No significant difference was observed for age (*p* = 0.672) or gender (*p* = 0.052) between ITP patients and controls. SNP genotypic frequencies, except for the NF-*κ*B-94ins/del ATTG genotype, were in the Hardy-Weinberg equilibrium in both patients and control groups.

The frequency of each genotype and allele for NLRP3 inflammasome genes in ITP patients and controls was listed in Table 3. Among the five detected SNPs of the NLRP3 inflammasome, only the distribution of NF-*κ*B-94ins/del ATTG was found significantly different between ITP patients and controls. Homozygote insertion of the wildtype (WW) genotype disclosed a statistically increased susceptibility of ITP when compared with the heterozygote (WD) genotype (OR = 2.003, 95% CI: 1.440–2.787, *p* < 0.001) or the homozygote deletion (DD) genotype (OR = 1.591, 95% CI: 1.061–1.368, *p* = 0.024). As for the frequency of the allele, the -94insATTG (W) allele was significantly higher in ITP cases compared to controls (62.53% versus 54.61%, *p* = 0.002). Moreover, the W allele was significantly associated with ITP susceptibility (OR = 1.387, 95% CI 1.126–1.708).

### 3.2. The Association between NF-*κ*B-94ins/del ATTG Polymorphism and Clinical Characteristics of ITP Patients

We evaluated the clinical relevance of NF-*κ*B-94ins/del ATTG polymorphisms in ITP patients (Table 4). The ITP patients were stratified in four clinical groups according to disease course, and no significant difference of the NF-*κ*B-94ins/del ATTG polymorphism was found between them. We also investigated the association between this polymorphism and therapeutic response in ITP. There was a marginally increased frequency of the WW genotype in the NR group than in CR + R group (*p* = 0.054). Moreover, ITP patients were also divided into sITP and nsITP according to the platelet count before treatment, but no significant difference was found.

**Figure 1:**
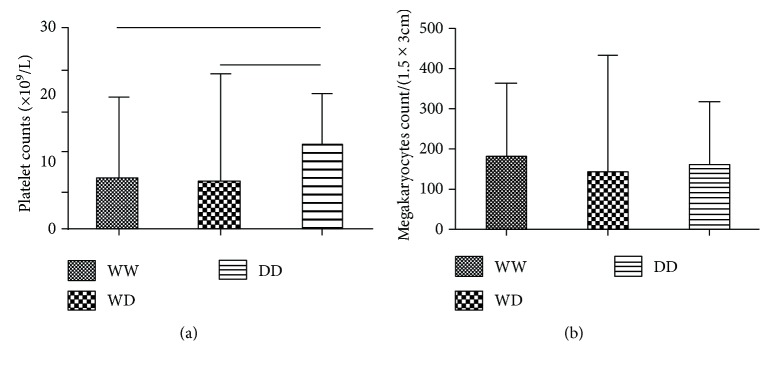
(a) The platelet counts of ITP patients with the WW genotype (7 × 10^9^/L) or WD genotype (7.5 × 10^9^/L) were lower than those with the DD genotype (12.5 × 10^9^/L) (*p* = 0.032). (b) As for megakaryocyte counts in ITP patients, no significant correlation was found among the three genotypes (*p* = 0.085).

Specifically, the platelet counts of ITP patients with the WW genotype (median 7 × 10^9^/L) or the WD genotype (median 7.5 × 10^9^/L) were lower than those with the DD genotype (median 12.5 × 10^9^/L, *p* = 0.032, Figure 1(a)). We further analyzed the association between the distribution of NF-*κ*B-94ins/del ATTG and megakaryocytes in ITP patients, and no correlation was found between them (*p* = 0.085, Figure 1(b)).

Our data showed that there was a significant difference (*p* = 0.037) in gender between patients with NF-*κ*B-94ins/del ATTG WW and the DD genotype. We also found that the frequency of the W allele was significantly higher in female than in male patients (65.32% versus 58.06%, *p* = 0.038). In addition, no statistical difference was found between genotype distribution and age of onset.

A total of 148 ITP patients were included to determine the antiplatelet autoantibodies against GPIIb/IIIa and GPIb/IX, including 69 patients for positive autoantibodies and 79 for negative autoantibodies. However, there was no significant correlation between the frequencies of NF-*κ*B-94ins/del ATTG and autoantibodies.

### 3.3. NF-*κ*B Gene Expression Was Downregulated and Correlated with NF-*κ*B-94ins/del ATTG Genotype in ITP Patients

**Figure 2:**
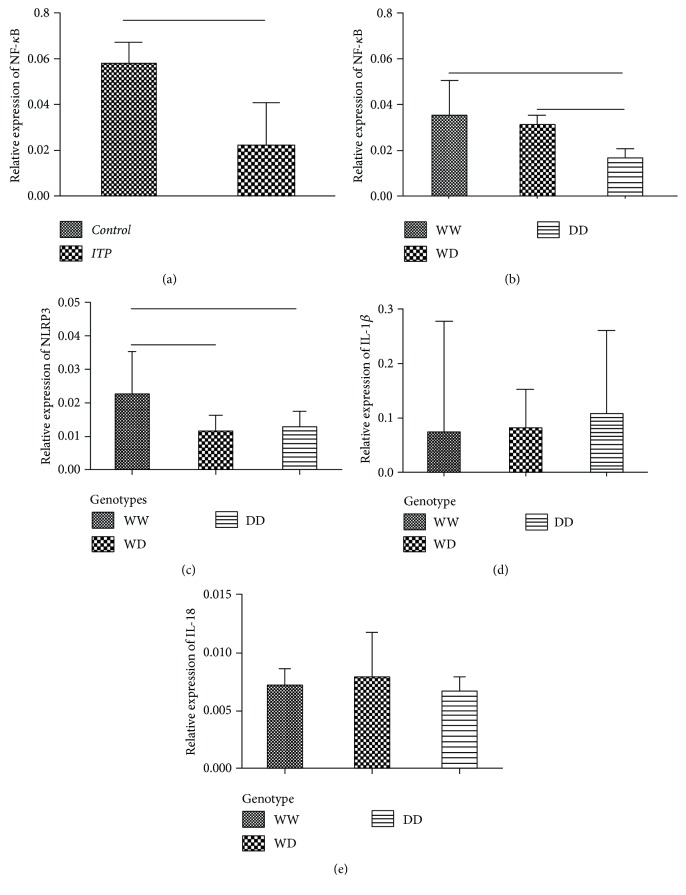
(a) Significantly lower NF-*κ*B mRNA levels were found in ITP compared with controls (*p* < 0.0001). (b) NF-*κ*B expression levels in ITP patients correlated with NF-*κ*B-94ins/del ATTG genotype status. (c) The expression of NLRP3 was also found significantly different in ITP patients with the WW genotype (median 0.023), WD genotype (median 0.011), and DD genotype (median 0.013, *p* = 0.045). (d, e) However, it showed no difference in IL-1*β* or IL-18 mRNA expression among the three genotypes.

A total of 40 ITP patients and 41 controls were used to determine the expression level of NF-*κ*B. A significantly lower NF-*κ*B mRNA level was found in ITP patients (median 0.0217) compared with controls (median 0.0575, *p* < 0.0001.  Figure 2(a)). Moreover, NF-*κ*B expression level in ITP patients correlated with NF-*κ*B-94ins/del ATTG genotype status. WW or WD genotype ITP patients displayed ~2.0-fold higher mRNA expression compared with DD individuals (median 0.030, 0.036, and 0.015, respectively; *p* = 0.033), which indicated a gene dose-dependent expression (Figure 2(b)).

With a similar trend, the expression of NLRP3 was also found significantly different in ITP patients with the WW genotype (median 0.023), WD genotype (median 0.011), and DD genotype (median 0.013, *p* = 0.045, Figure 2(c)). However, it showed no difference of IL-1*β* or IL-18 mRNA expression among the three genotypes (Figures 2(d) and 2(e)).

### 3.4. Th17 Was Correlated with NF-*κ*B-94ins/del ATTG Genotypes

**Figure 3:**
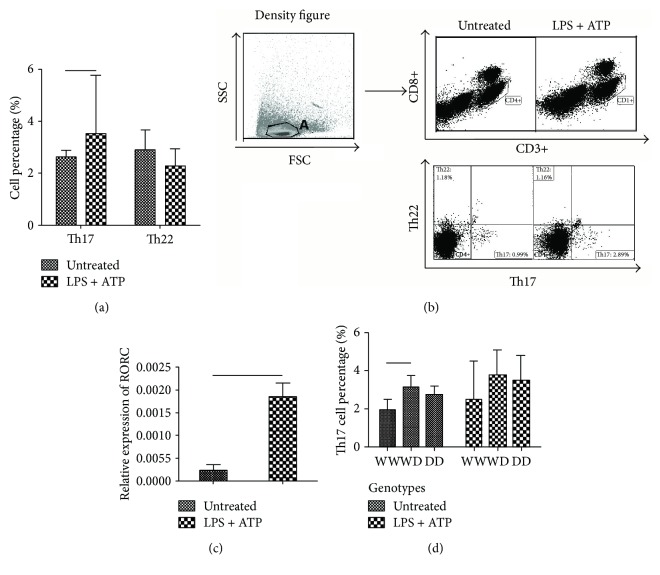
(a, b) Th17 cells were significantly increased after the NLRP3 inflammation activation (median, 3.51%; 1.00–10.68%) compared to without activation (median, 2.76%; 0.45–4.17%; *p* = 0.023), while no statistical difference was found for the percentage of Th22 cells (*p* = 0.173). (c) RORC expression was higher in the NLRP3 activation group of ITP patients (0.001845) compared with untreated ones (0.0002345; *p* = 0.012). (d) The percentage of Th17 cells for WW, WD, or DD were 1.94%, 3.23%, or 2.76% (*p* = 0.042) in the untreated group and 2.48%, 5.78%, or 3.51% after being stimulated (*p* = 0.405).

Based on NF-*κ*B-94ins/del ATTG genotypes, Th17 percentage in untreated PBMCs from ITP patients was significantly different between WW and WD genotypes (*p* < 0.05, Figure 3(d)). However, the statistical differences between the three groups disappeared after the NLRP3 inflammasome activation. The percentages of Th17 cells for the WW, WD, and DD genotypes were 1.94%, 3.23%, and 2.76% (*p* = 0.042) in the untreated group and 2.48%, 3.78%, and 3.51% after being stimulated (*p* = 0.405, Figure 3(d)).

### 3.5. The Activation of NLRP3 Inflammasome-Upregulated Th17 Cells through Increasing RORC Expression in ITP Patients

As our previous work demonstrated the upregulated Th17 as well as Th22 cells in ITP patients, we next analyzed the shift of Th17 and Th22 cells in PBMCs of ITP patients after activating the NLRP3 inflammasome with LPS followed by ATP. We found that Th17 cells were significantly increased after the NLRP3 inflammasome activation (LPS + ATP, median 3.51%, 1.00%–10.68%) than without activation (untreated, median 2.76%, 0.45%–4.17%, *p* = 0.023), while no statistical difference was found for the percentage of Th22 cells (*p* = 0.173) (Figures 3(a) and 3(b)).

To further determine whether the increased Th17 level after the NLRP3 inflammasome activation with LPS and ATP was correlated with RORC, we analyzed RORC mRNA expression by RT-PCR. We identified a significantly positive correlation between Th17 percentage and its transcription factor, RORC. The results showed that RORC expression was higher in the NLRP3 activation group of ITP patients (median 0.001845) compared with unstimulated group (median 0.0002345, *p* = 0.012, Figure 3(c)). The expression of AHR had no statistical alteration after NLRP3 activation in ITP patients compared with controls (*p* = 0.441).

### 3.6. The Influence of NLRP3 Activation on Apoptosis of PBMCs in ITP Patients

**Figure 4:**
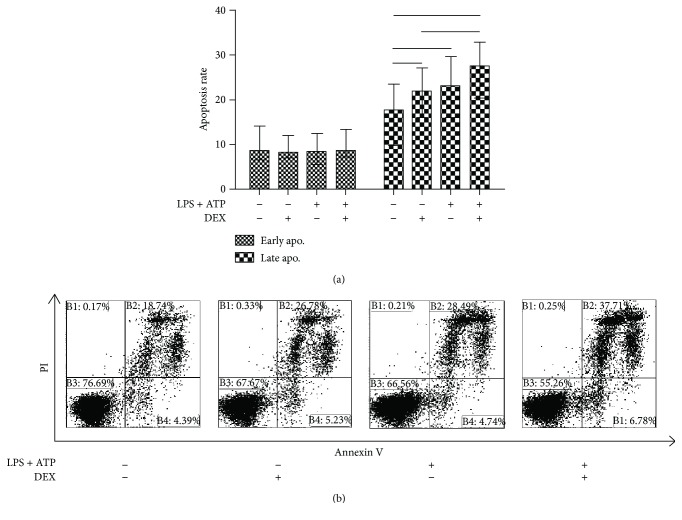
(a, b) The frequency of late apoptosis was significantly increased after NLRP3 inflammasome activation or treatment with DEX.

To explore the function of NLRP3 in ITP, we detected the apoptosis of PBMCs after being activated with LPS and ATP or treated with DEX. However, no significant difference of early apoptosis was found after NLRP3 inflammasome activation with or without 10 mmol/L DEX in vitro. However, increased late apoptosis was observed after NLRP3 inflammasome activation (median 23.13%, 7.09%–47.15%, *p* = 0.023) or being treated with 10 mmol/L DEX (median 22.015%, 7.94%–45.66%, *p* = 0.023) compared to the control (median 17.67%, 5.36%–25.07%). Furthermore, in comparison with being treated with DEX alone, further increases of late apoptosis were found when PBMCs of ITP were incubated in DEX combined with NLRP3 inflammasome activation (median 27.7%, 9.77%–86.61%, *p* = 0.041). However, there was no significant difference between NLRP3 inflammasome activation alone and the combine of DEX and inflammasome activation (*p* = 0.071) (Figures 4(a) and 4(b)).

## 4. Discussion

ITP is an immune-mediated acquired disorder characterized by a transient or persistent decrease in platelet count due to decreased production and increased peripheral destruction of platelets [27]. There are many genetic predisposing factors in the pathogenesis of ITP [29, 30]. A recent study has demonstrated that the LRP3 inflammasome plays a critical role in the pathogenesis of many autoimmune diseases. However, little was known about the clinical relevance and function of NLRP3 gene polymorphisms in ITP. For the first time, our study uncovered the associations between gene polymorphisms involved in the NLRP3 inflammasome and clinical features of ITP in a Northern Han Chinese population.

NF-*κ*B, a ubiquitous transcription factor, has been found to play important roles in the activation and function of the NLRP3 inflammasome [31]. NF-*κ*B was reported to regulate the expression of many genes involved in angiogenesis, cell adhesion, proliferation, differentiation, and apoptosis [32, 33]. The NF-*κ*B-94-ins/del ATTG polymorphism is located in the NF-*κ*B gene promoter region. A meta-analysis [34] demonstrated that the NF-*κ*B D allele decreased the risk of autoimmune and inflammatory diseases, which might be due to the lower promoter activity and binding affinity of NF-*κ*B with the D allele leading to low expression levels of the NF-*κ*B protein. Many other studies have also suggested that the NF-*κ*B-94ins/del ATTG polymorphism may be implicated in the pathogenesis of human autoimmune and inflammatory diseases, including Graves' disease, UC, and psoriasis vulgaris [15–17]. Furthermore, Yalcin et al. demonstrated that the -94ins/del ATTG promoter polymorphism of NF-*κ*B may have functional consequences in Behchet's disease (BD) [35]. In the current study, we found that the W allele was more frequent in ITP patients than in controls. Similarly, the frequency of the homozygous (WW) genotype was also demonstrated to be higher. Our results indicated that the W allele and WW genotype can result in increased ITP susceptibility for individuals in ethnic Northern Han Chinese and might be used as a marker for ITP development. We also found that more of the WW genotype was carried by the female ITP patients. It suggests that sex hormones may play a role in the gender-related predominance. The increased NF-*κ*B gene expression associated with the W allele was reported to result in a decrease in p50/p65 heterodimers, the major activated form of NF-*κ*B [15]. Our results confirmed a high expression level of NF-*κ*B in ITP patients carrying the W allele, no matter whether they be WW or WD genotypes. Therefore, the SNP of the NF-*κ*B gene could influence the mRNA level of NF-*κ*B. Bauernfeind et al. demonstrated that LPS stimulation could lead to a strong and NF-*κ*B-dependent increase of NLRP3 mRNA expression, and signals provided by NF-*κ*B activators were necessary for NLRP3 activation in mouse macrophages [31]. Similar with a previous study [9], we also observed a significantly higher level of NLRP3 in newly-diagnosed ITP patients compared to healthy control. More importantly, we found a significantly increased expression of NLRP3 in ITP patients with the WW genotype than in those with the DD genotype, which indicated that the NLRP3 inflammasome may participate in ITP development. Hence, we may infer that increased activation of NF-*κ*B may promote the development of ITP by the NLRP3 inflammasome in individuals with the W allele.

Furthermore, we determined the relationships between the NF-*κ*B-94ins/del ATTG polymorphism and clinical characteristics of ITP patients. We found that the platelet counts of individuals with the W allele were lower than in those with the homozygote DD. However, no significant difference was found among the genotypes on megakaryocyte count, disease severity, or drug response. There is no sufficient evidence that the NF-*κ*B-94ins/del ATTG polymorphism can be regarded as the prognostic factor. Moreover, the frequency of the W allele was significantly greater in female ITP patients compared to male ITP patients, and its potential mechanism needs to be clarified in the future. Platelet production inhibition mediated by antiplatelet antibodies is a well-known mechanism causing low platelet counts in ITP [36]. However, there was no significant difference to be found between the NF-*κ*B-94ins/del ATTG polymorphism and antiplatelet GP IIb/IIIa and GP Ib/IX autoantibodies.

In addition, NF-*κ*B was crucial for the development and activation of Th cells and the disorder of NF-*κ*B could lead to impaired T cell function [37]. T-lymphocyte abnormalities are considered important in the pathogenesis of ITP. Our previous research reported that Th17 and Th22 cells were significantly higher in ITP patients than in healthy controls. In the present study, we found that the percentage of Th17 cells was significantly higher after activating the NLRP3 inflammasome with LPS followed by ATP activation in PBMCs of ITP patients. Furthermore, RORC, the transcription factor of Th17 cells, was higher in the NLRP3 activation group, which implied that the NLRP3 inflammasome was involved in the regulation of Th subsets in ITP. In the meantime, we found that Th17 percentage in WW genotype patients was lower than that in WD genotype patients. However, the difference disappeared after we activated the NLRP3 inflammasome. This indicated that one of the functions of NLRP3 is the regulation of Th17 responses that can markedly affect T cell-mediated autoimmunity. In addition, signals provided by NF-*κ*B activators are necessary for NLRP3 inflammasome activation to produce active IL-1*β* and IL-18 [31]. The NLRP3 inflammasome also potentially creates a microenvironment for Th17 cell differentiation [38, 39]. Thus, the NLRP3 inflammasome regulated by the NF-*κ*B pathway, at least partially, may take part in ITP pathogenesis mediated by the dysfunction of T helper cells. However, there are some inconsistencies among the genotypes, expression of NF-*κ*B, and Th17 percentages, which need further investigation in the future.

Recently it has been proven that NLRP3 (Q705K) was associated with an increased risk of developing CD in Swedish men [40]. However, no AA and CA genotypes of NLRP3 (rs35829419) were found in ITP patients and controls in our research. Although the IL-1*β* (rs16944) polymorphism was reported to be associated with the risk of many autoimmune diseases [22–24], our data indicated that it did not influence susceptibility to ITP. In accordance with a previous study [41], our results showed that the IL-18 promoter -607 A/C polymorphism was almost equally distributed between ITP patients and the controls, which indicated that it may not be used as a stratification marker to predict the susceptibility to Chinese ITP.

Many studies have uncovered the performance of the CARD8 gene polymorphism rs2043211 in autoimmune disorders, RA, CD, and IBD [13, 14, 42, 43]. However, these findings were varied and even contradictory. For example, many studies on the association between the CARD8 (rs2043211) and CD susceptibility are quite inconsistent. A previous study reported that T allele has a protective effect in CD patients [14, 43], while another study found that it increased the risk for developing CD [42]. However, in our study, no significant difference of both allele and genotype of CARD8 was found in ITP patients and controls. In consideration of the different study backgrounds, CARD8 (rs2043211) may behave differently in various kinds of diseases, which may be associated with different geographical distributions. Therefore, the rs2043211 gene polymorphism should be further studied in other regions.

NLRP3 inflammasome activation in PBMCs drives cell death, which may be related to the Caspase-1-induced pyroptosis [44]. More importantly, the combination of NLRP3 inflammasome activation and dexamethasone further contributes to the cell apoptosis of PBMCs, which may inform us that NLRP3 inflammasome activation may enhance the immunosuppression of dexamethasone in ITP therapy.

In conclusion, the NF-*κ*B-94ins/del ATTG genotype contributes to the susceptibility and imbalanced Th17 cells in patients with immune thrombocytopenia. The NF-*κ*B-94ins/del ATTG genotype might serve as a novel biomarker and potential target for ITP.

## Figures and Tables

**Table 1:** Clinical characteristics of ITP patients and controls.

Parameters	ITP (*n* = 403)	Controls (*n* = 336)
Gender (male : female)	155 : 248	153 : 183
Age (years, median, range)	42 (14–82)	41 (16–76)
Stage		
Newly diagnosed ITP	203	
Persistent ITP	61	
Chronic ITP	136	
Refractory ITP	3	
Severity		
Severe ITP	226	
Nonsevere ITP	177	
Response to treatment		
CR	161	
R	182	
NR	60	

**Table 2:** The primers for PCR.

Gene name	Forward primer	Reverse primer
NF-*κ*B	5′-TCC AGA CCA ACA ACA ACC CC-3′	5′-GAT CTT GAG CTC GGC AGT GT-3′
NLRP3	5′-CAG ACT TCT GTG TGT GGG ACT GA-3′	5′-TCC TGA CAA CAT GCT GAT GTG A-3′
IL-1*β*	5′-GCC CTA AAC AGA TGA AGT GCT C-3′	5′-GAA CCA GCA TCT TCC TCA G-3′
IL-18	5′-GCT TGA ATC TAA ATT ATC AGT C-3′	5′-GAA GAT TCA AAT TGC ATC TTA T-3′
RORC	5′-CAA TGG AAG TGG TGC TGG TTA G-3′	5′-GGG AGT GGG AGA AGT CAA AGA T-3′
AHR	5′-CAA ATC CTT CCA AGC GGC ATA-3′	5′-CGC TGA GCC TAA GAA CTG AAA G-3′
GAPDH	5′-GCT CTC TGC TCC TCC TGT TC-3′	5′-GTT GAC TCC GAC CTT CAC CT-3′

**Table 3:** Genotype and allele distribution of NLRP3 gene polymorphisms.

Polymorphisms	ITP *n* (%)	Controls *n* (%)	OR (95% CI)	*P* value
NF-*κ*B-94ins/delATTG				
Genotype				
WW	179 (44.42)	101 (30.06)		
WD	146 (36.23)	165 (49.11)	2.003 (1.440–2.787)	0
DD	78 (19.35)	70 (20.83)	1.591 (1.061–1.368)	0.024
Allele				
W	504 (62.53)	367 (54.61)		
D	302 (37.47)	305 (45.39)	1.387 (1.126–1.708)	0.002
CARD8 (rs2043211)				
Genotype				
AA	98 (24.32)	102 (30.36)		
AT	206 (51.12)	152 (45.24)	0.709 (0.501–1.004)	0.052
TT	99 (24.56)	82 (24.40)	0.796 (0.532–1.191)	0.267
Allele				
A	402 (49.88)	356 (52.98)		
T	404 (50.12)	316 (47.02)	0.883 (0.720–1.084)	0.235
IL-18 (rs1946518)				
Genotype				
GG	123 (30.52)	90 (26.79)		
GT	184 (45.66)	175 (52.08)	1.3 (0.924–1.829)	0.132
TT	96 (23.82)	71 (21.13)	1.011 (0.671–1.523)	0.959
Allele				
G	430 (53.35)	355 (52.83)		
T	376 (46.65)	317 (47.17)	1.021 (0.832–1.254)	0.841
IL-1*β* (rs16944)				
Genotype				
GG	121 (30.02)	81 (24.11)		
GA	183 (45.41)	167 (49.7)	1.363 (0.960–1.936)	0.083
AA	99 (24.57)	88 (26.19)	1.328 (0.888–1.985)	0.166
Allele				
G	425 (52.73)	329 (48.96)		
A	381 (47.27)	343 (51.04)	1.163 (0.947–1.427)	0.149
NLRP3 (rs35829419)				
Genotype				
AA	0 (0)	0 (0)		
CA	0 (0)	0 (0)		
CC	403 (100)	336 (100)		
Allele				
A	0 (0)	0 (0)		
C	806 (100)	672 (100)		

**Table 4:** The results of NF-*κ*B-94ins/del and clinical characteristics.

	Genotypes			Allelic frequency	
	WW	WD	DD	Allele W	Allele D
Gender					
Male	62 (40%)	56 (36.13%)	37 (23.87%)	180 (58.06%)	130 (41.94%)
Female	117 (47.18%)	90 (36.29%)	41 (16.53%)	324 (65.32%)	172 (34.68%)
*P*		0.282	0.037		0.038
Stage					
nITP	95 (46.8%)	72 (35.47%)	36 (17.73%)	262 (64.53%)	144 (35.47%)
pITP	27 (44.26%)	20 (32.79%)	14 (22.95%)	74 (60.66%)	48 (39.34%)
cITP	57 (41.91%)	52 (38.24%)	27 (19.85%)	166 (61.03%)	106 (38.97%)
rITP	0	2	1	2	4
*P*		0.396	0.379		0.351
Severity					
sITP	76 (42.94%)	59 (33.33%)	42 (23.73%)	211 (59.60%)	143 (40.40%)
nsITP	103 (45.58%)	87 (38.50%)	36 (15.93%)	293 (64.82%)	159 (35.18%)
*P*		0.398	0.061		0.129
Response					
CR + R	149 (43.44%)	122 (35.57%)	72 (20.99%)	420 (94.17%)	26 (5.83%)
NR	30 (50.00%)	24 (40.00%)	6 (10.00%)	84 (70.00%)	36 (30.00%)
*P*		0.938	0.054		0.067
MAIPA					
Positive	26 (37.68%)	25 (36.23%)	18 (26.09%)	77 (55.80%)	61 (44.20%)
Negative	37 (46.84%)	23 (29.11%)	19 (24.05%)	97 (59.88%)	65 (40.12%)
*P*		0.257	0.473		0.476

## Data Availability

The data used to support the findings of this study are available from the corresponding author upon request.
